# Botulinum toxin as a promising surgical strategy for chronic anal fissure: do the dose and injection site matter? Comparison of doses and injection sites of botulinum toxin for chronic anal fissure: A systematic review and network meta-analysis of randomized controlled trials

**DOI:** 10.1097/JS9.0000000000000022

**Published:** 2023-03-03

**Authors:** Hao Xu, Wei Yang, Zubing Mei

**Affiliations:** a Department of Anorectal Surgery, Shuguang Hospital, Shanghai University of Traditional Chinese Medicine; b Anorectal Disease Institute of Shuguang Hospital; c Department of Anorectal, Shanghai Fourth People’s Hospital, School of Medicine, Tongji University, Shanghai, China

Chronic anal fissure (CAF) often shows a considerable reluctance to heal for the hypertonia and spasm of the internal sphincter. CAF pain is very intense and sharp and always described as tearing, knife-cut or passing broken glass. Lateral internal sphincterotomy remains the preferred treatment modality of all surgical options with high healing rates ranging from 88 to 100%, followed by incontinence rates ranging from 8 to 30% which can have a significant impact on a person’s quality of life.^1^ Botulinum toxin (BT) has been widely used in the treatment of CAF due to its ability to promote healing and lower risk of incontinence. However, there is still no consensus on the BT dose and injection site which are associated with the curative effect. Vitoopinyoparb *et al.* 2 performed a systematic review and network meta-analysis of randomized controlled trials and identified that low-dose (≤20 U) BT was optimal, and injection out of the fissure site improved short-term outcomes while injection each side of the fissure site tended to reduce recurrence in the long run. It is of great interest to provide a relatively standard protocol to guide anorectal surgeons in the management of BT.

BT, as a temporary chemical sphincterotomy, can lead to the blockade of acetylcholine release and cause short-term paralysis of internal sphincter muscle to reduce the anal tone. Based on our clinical experience, the higher the BT dose (within 100 U in general), the higher the healing rate of CAF. According to the survey conducted by the American Society of Colon and Rectal Surgeons, dose above 50 U of BT appeared to correlate with higher success rate and healing rate.^3^ Maria *et al.* 4 has confirmed that higher doses can improve the healing rate without increasing complications or side effects. However, a meta-analysis including 18 clinical trials concluded that the healing rate decreased with the increase of the BT dose (*P*=0.048), but the incontinence rate (*P*=0.048) and recurrence rate (*P*=0.002) increased slightly with the increase of the BT dose^5^. Another meta-analysis involving 1577 patients from 34 prospective studies showed that the healing rates ranged from 33 to 96% the BT with the reported BT doses ranging from 5 to 150 IU across the analyzed studies. However, there was no correlation between postoperative healing rate and BT dose (*P*=0.0708), and the rates of postoperative complications and fecal incontinence were not found to be in a dose-dependent manner.^6^ Conflicts over BT dose selection often cause confusion among anorectal surgeons. In this network meta-analysis, Vitoopinyoparb and colleagues found that low-dose (≤20 U) BT was optimal based on 27 randomized controlled trials, providing high-level evidence on this regard.

Based on the existing knowledge, most studies recommend injecting BT into internal sphincter in three or four quadrants around the anal canal. We also believe that BT should be injected into the internal sphincter because the fundamental pathogenic is hypertonia and spasm of the internal sphincter in CAF. As Brisinda *et al.* 4 said, the internal sphincter is readily visible and easier to inject than the external sphincter. To improve the healing rate, we usually inject the BT both outside the fissure and on each side of the fissure. A study which included 50 CAF patients showed that injections on both sides of the anterior midline were more effective than those on the posterior midline.^7^ In this study, the short-term healing rate of external injection was better than that of both sides of the fissure, while the long-term recurrence and incontinence of bilateral injection were less.^1^

In general, we believe these results of this study have high value for clinical application, and large multicenter randomized controlled studies should be advocated to further confirm the findings.

## Ethical approval

Not applicable.

## Sources of funding

This study is funded by the Shenkang Clinical Three-Year Action Plan (major research) (grant no. SHDC2020CR2007A), and the National Natural Science Foundation of China (81774112).

## Authors’ contribution

H.X.: design and write the commentary. W.Y.: review the commentary. Z.M.: design, review, and revise the commentary.

## Conflicts of interest disclosure

The authors declare that they have no financial conflict of interest with regard to the content of this report.

## Research registration unique identifying number (UIN)

None.

## Guarantors

Zubing Mei, Hao Xu.

## References

[1] Vogel JD Johnson EK Morris AM . Clinical practice guideline for the management of anorectal abscess, fistula-in-ano, and rectovaginal fistula. Dis Colon Rectum 2016;59:1117–1133. 27824697 10.1097/DCR.0000000000000733

[2] Vitoopinyoparb K Insin P Thadanipon K . Comparison of doses and injection sites of botulinum toxin for chronic anal fissure: a systematic review and network meta-analysis of randomized controlled trials. Int J Surg 2022;104:106798. 35934283 10.1016/j.ijsu.2022.106798

[3] Borsuk DJ Studniarek A Park JJ . Use of botulinum toxin injections for the treatment of chronic anal fissure: results from an American Society of Colon and Rectal Surgeons Survey. Am Surg 2021:31348211023446. 10.1177/00031348211023446 34092078

[4] Brisinda G Cadeddu F Mazzeo P . Botulinum toxin A for the treatment of chronic anal fissure. Expert Rev Gastroenterol Hepatol 2007;1:219–228. 19072412 10.1586/17474124.1.2.219

[5] Lin JX Krishna S Su’a B . Optimal dosing of botulinum toxin for treatment of chronic anal fissure: a systematic review and meta-analysis. Dis Colon Rectum 2016;59:886–894. 27505118 10.1097/DCR.0000000000000612

[6] Bobkiewicz A Francuzik W Krokowicz L . Botulinum toxin injection for treatment of chronic anal fissure: is there any dose-dependent efficiency? A meta-analysis. World J Surg 2016;40:3064–3072. 27539490 10.1007/s00268-016-3693-9 PMC5104788

[7] Maria G Brisinda G Bentivoglio AR . Influence of botulinum toxin site of injections on healing rate in patients with chronic anal fissure. Am J Surg 2000;179:46–50. 10737578 10.1016/s0002-9610(99)00255-x

